# Overview of the Role of Cell Wall DUF642 Proteins in Plant Development

**DOI:** 10.3390/ijms20133333

**Published:** 2019-07-06

**Authors:** José Erik Cruz-Valderrama, Ximena Gómez-Maqueo, Alexis Salazar-Iribe, Esther Zúñiga-Sánchez, Alejandra Hernández-Barrera, Elsa Quezada-Rodríguez, Alicia Gamboa-deBuen

**Affiliations:** 1 Instituto de Ecología, Universidad Nacional Autónoma de México. Mexico City 04510, Mexico; 2 Universidad Nacional Autónoma de México, Mexico City 04510, Mexico

**Keywords:** DUF642 family, homogalacturonans, plant cell wall, plant development, pectins

## Abstract

The DUF642 protein family is found exclusively in spermatophytes and is represented by 10 genes in Arabidopsis and in most of the 24 plant species analyzed to date. Even though the primary structure of DUF642 proteins is highly conserved in different spermatophyte species, studies of their expression patterns in Arabidopsis have shown that the spatial-temporal expression pattern for each gene is specific and consistent with the phenotypes of the mutant plants studied so far. Additionally, the regulation of DUF642 gene expression by hormones and environmental stimuli was specific for each gene, showing both up- and down-regulation depending of the analyzed tissue and the intensity or duration of the stimuli. These expression patterns suggest that the DUF642 genes are involved throughout the development and growth of plants. In general, changes in the expression patterns of DUF642 genes can be related to changes in pectin methyl esterase activity and/or to changes in the degree of methyl-esterified homogalacturonans during plant development in different cell types. Thus, the regulation of pectin methyl esterases mediated by DUF642 genes could contribute to the regulation of the cell wall properties during plant growth.

## 1. Introduction

Plant cell walls are dynamic compartments whose composition and chemical structure vary during plant development in response to environmental signals. Cell walls are composed of three layers: the middle lamella, and the primary and secondary cell walls. Primary cell wall deposition continues through cell growth and expansion. It is a very complex and dynamic structure composed mainly of three types of polysaccharides: cellulose, hemicelluloses, and pectins. Cellulose microfibrils and hemicelluloses constitute a network with structural proteins that are embedded in a gel-like matrix of pectins. Localization of different classes of polysaccharides within the cell wall appears to depend on species, organ, tissue, and cell type, and the pectin network is temporally and spatially regulated during plant development. Alterations of the cell wall structure and changes in properties during plant development are mainly the result of protein activities. Enzymatic activity and protein interactions respond to developmental and environmental signals and tightly regulate the complex and dynamic structure of plant cell walls.

In terrestrial plants, the chemical structure of homogalacturonans (HGs), a type of pectin that is comprised of chains of α-1,4-galacturonic acid, is modified by de-methyl-esterification processes during plant development by pectin methyl esterases, and pectin methyl esterase inhibitory proteins regulate the degree of HGs’ esterification of the cell wall in different cell types. Consequently, cell or tissue-specific function could be influenced by the HGs’ status [1]. An increase in complexity in the regulation of HGs’ esterification status through plant evolution has been described including a diversification of the gene families involved either in pectin synthesis or in pectin modification [2,3]. The shift from an aquatic to a terrestrial environment constitutes a milestone in the complexity of pectin regulation. In the algae *Chara*, cell growth is a result of a calcium pectate cycle that does not involve enzymatic activities [4]. Charophytes are the closest related group to land plants, thus, this enzyme-less mechanism could be still present in terrestrial plants [5].

The DUF642 protein family is found exclusively in spermatophytes and is involved in regulating HGs esterification. The DUF642 family was first described in apoplast proteomes of rosette leaves in *Arabidopsis thaliana* (Arabidopsis) by Boudart et al. [6] and subsequently in cell wall proteomes from different tissues and plant species from both dicotyledons and monocotyledons [7,8]. This multigene family is represented by 10 genes in Arabidopsis as well as in other plant species [9]. DUF642 proteins contain two DUF642 domains (PF04642) that present two conserved motifs NGXFEXXP and CGPVXD, determined as the family signature. A phylogenetic analysis revealed four clades of orthologous genes for all spermatophytes (Clades A, B, C, and D). After the divergence of gymnosperms and angiosperms, a duplication of Clade A occurred, producing two subclades (A1 and A2), and a subsequent duplication occurred after the divergence of monocotyledons and eudicotyledons [9].

The DUF642 proteins are encoded by genes that contain three exons. The first exon encodes a signal peptide, and the other two exons encode the DUF642 domains. Proteomic and cellular localization studies of the Clade A proteins indicated they were located primarily in the cell wall [6,10]. However, the protein encoded by *At3g08030* was located in the nuclear pore, probably because this gene has an alternative first exon edition so one of the possible encoded protein lacks the signal peptide. The proteins in Clades B, C, and D have a GPI-anchor site at the carboxyl terminus and were detected in cell membrane proteomes [11].

The interactions of the DUF642 proteins with polysaccharides and cell wall enzymes have been demonstrated in vitro. The protein encoded by *At3g08030* (Clade A2) interacted specifically with cellulose [11]. The proteins encoded by *At4g32460*/*BDX* and *At5g11420* (Clade A1) interacted with the catalytic domain of a pectin methyl esterase (PME) [12]. In two transgenic Arabidopsis lines overexpressing *BDX* the PME activity increased in the seeds, seedlings, and the apical meristems, but no changes in PME activity were detected in the leaves [13]. PME activity was higher in the seeds of both overexpressing lines than it was in the control seeds during germination [13,14]. PME activity was also higher in seedlings, stems, leaves, and fruits in Arabidopsis plants overexpressing the *VqDUF642* gene (Clade A1) of *Vitis quinquangularis* [15]. The leaves of Arabidopsis plants overexpressing the *AhDGR2* gene (Clade A1) of *Amaranthus hypochondriacus* showed a decrease in PME activity, whereas an increase in PME activity was detected in the roots [16]. The phenotypes of all the Clade A1 protein mutants in Arabidopsis have been established. The *At5g25460/dgr2* mutant had a shorter root and smaller rosette leaves than the wild-type [17]. No alterations in the phenotypes of the *At5g11420* and *At1g80240/dgr1* mutants compared with the wild-type have been described. The *At4g32460*/*bdx* mutants had shorter siliques, seeds with altered development, and longer hypocotyl than the wild-type [18]. Among Arabidopsis Clade A2 mutants, the *At2g41800/teb* mutant is the only one for which a phenotype (longer hypocotyls) has been established [19]. No phenotypes have been established for the two Clade B genes *At1g29980* and *At2g34510*, or the Clade D gene *At5g14150*.

## 2. Expression Patterns of *DUF642* Genes

**Figure 1:**
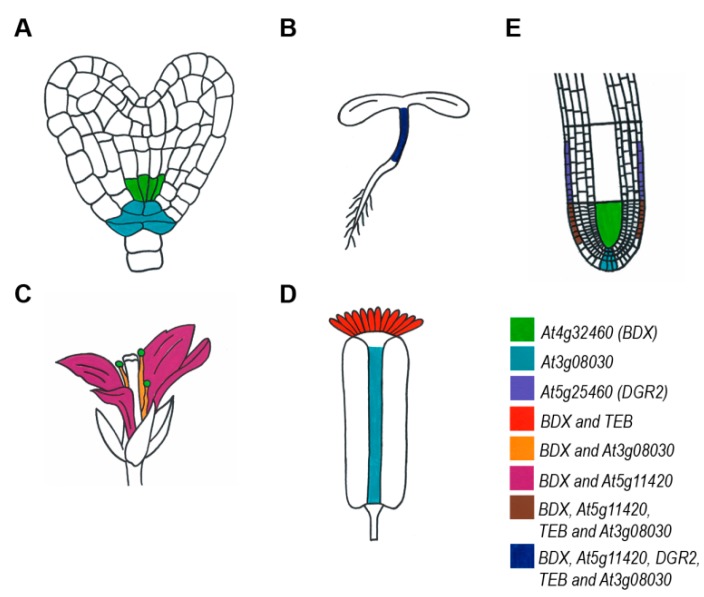
Expression patterns of the *DUF642* genes described to date using reporter genes during Arabidopsis plant development.

The expression patterns of the Clade A *DUF642* genes have been studied in Arabidopsis plants transformed with constructs containing the putative promoter region fused to the reporter genes *ER-GFP* and *GUS* (Figure 1). Differential expression patterns were detected at different stages of plant development (Table 1).

In Arabidopsis during embryonic development, *BDX* and *At3g08030* were expressed from the heart stage onwards. In the late heart stage, *At3g08030* was expressed in the quiescent center and in the columella cells, while *BDX* was expressed in the vascular tissue (Figure S1) [13].

During seedling development in Arabidopsis, *BDX*, *At5g11420*, *DGR2*, *TEB*, and *At3g08030* were expressed in hypocotyls and *BDX* and *DGR2* were also expressed in cotyledons, but no expression was detected for *At5g11420*, *TEB*, and *At3g08030* [13,17,20]. In hypocotyls, specific expression of *BDX*, *At5g11420*, *At3g08030*, and *TEB* in the epidermal cells was determined by confocal microscopy. *DGR1* and *At2g34510* expression was detected specifically in hypocotyls grown under shade conditions [21]. Transcriptomic studies in *Brassica rapa* indicated that three orthologous genes of *BDX*, *At5g11420*, and *DGR2* were expressed in hypocotyls [20].

The expression patterns of only *DGR1*, *BDX*, and *DGR2* have been studied in leaves of Arabidopsis. *BDX* expression was localized in the vascular tissue, *DGR2* expression was located in the leaf primordium, and *DGR1* expression was not detected in the leaves [13,17].

In flowers of Arabidopsis, *BDX* was expressed in petals, vascular tissue of the filament, anther, and stigmatic papilla. *At5g11420* was expressed in petals and abscission zones of floral whorls, *TEB* was expressed only in stigmatic papilla, and *At3g08030* was expressed in the replum of androecium and epidermis of the filaments of stamens [13,18] (Figure S1).

In Arabidopsis, *DGR1*, *BDX*, *At5g11420*, *DGR2*, *TEB*, and *At3g08030* expression was detected in the primary root. *BDX*, *At5g11420*, *DGR2*, *TEB*, and *At3g08030* were expressed in epidermal cells of the meristematic region. *BDX* also was expressed in vascular tissue and *At3g08030* was expressed in the quiescent center and in columella [13,17,18] (Figure S1). These expression patterns were similar to those found during embryonic development.

**Figure 2:**
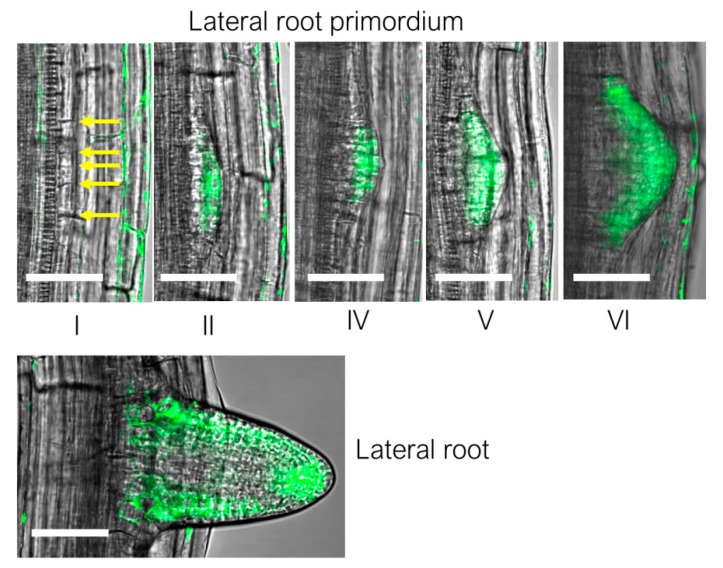
Expression patterns of *At3g08030* during lateral root development. The Arabidopsis *pAt3g08030::ER-GFP-*transgenic plants were obtained as described in Salazar-Iribe et al. [19]. Roman numerals indicate the developmental stages of the lateral root initiation process. The images are all section images. n = 8–10 roots for each developmental stage. Scale bars = 20 µm.

During development of the lateral roots, *BDX* and *At3g08030* expression was detected from stage II onwards, when cell proliferation begins (Figure 2). Further, at the moment of radicle protrusion, *At5g11420* and *TEB* expression was detected in the epidermal cells that surround the radicle protrusion zone. The expression patterns of *DGR1* and *DGR2* during this process have not been determined.

The expression patterns of the Clade A *DUF642* genes were altered by plant hormones (Table 1 and Table S1). For example, auxins and gibberellins increased the expression levels of *BDX* and *TEB* during Arabidopsis seed germination [22]. Exogenous auxins and gibberellins also altered the expression pattern of *BDX* by promoting its expression in cortex cells in addition to vascular tissue of Arabidopsis. The expression pattern of *At3g08030* was not altered by the addition of auxins [13].

## 3. Subcellular Localization of DUF642 Proteins

DUF642 proteins are very abundant in cell wall proteomes from different tissues and plant species [7,8]. The At3g08030 protein has been found in all Arabidopsis cell wall proteomes reported so far. TEB and DGR1 were found in the proteomes of cell suspension cultures, whereas DGR2 was found in the proteomes of cell suspension cultures, hypocotyls, and mature stems. The At5g11420 protein was present in the proteomes of apoplast, hypocotyls, and mature stems, whereas BDX was found only in the mature stem cell wall proteome [28]. The localization of BDX and TEB in the Arabidopsis cell wall was confirmed by confocal microscopy in hypocotyl cells [18,19]. The VqDUF642 protein was localized in the cell wall of tobacco epidermal cells [15] and peach PpDU642 (TEB) was localized in the cell wall and extracellular space during a transient expression in tomato fruit [29].

**Figure 3:**
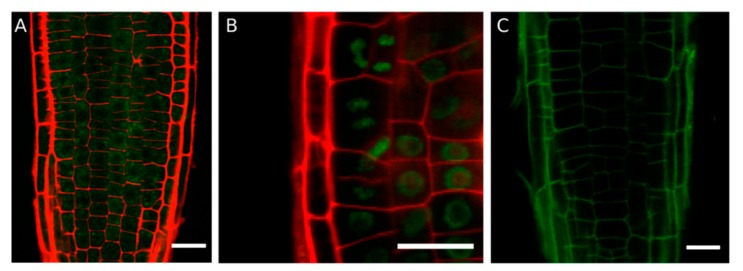
Subcellular localization of the TEB protein in meristematic root cells synchronized with hydroxyurea. (**A**) Arabidopsis *pTEB::TEB-GFP* seedlings were treated with hydroxyurea for 16 h, and a diffuse pattern of GFP in meristematic cells of the cortex was observed. The cell wall was stained with propidium iodide (red). (**B**) After 18 h of hydroxyurea treatment, GFP was detected as a ring surrounding the nucleus in the cortex cells. (**C**) TEB was located in the cell wall after 19 h of hydroxyurea treatment. Hydroxyurea stops DNA synthesis and after 16–17 h most of the cells are at the G2/M transition and at 18–20 h cytokinesis occurs according to Cools et al. [31]. Seedlings were sown in MS medium and transferred on day 6 to MS medium with hydroxyurea for 16–20 h. The images are all section images. Scale bars = 20 µm. The Arabidopsis transgenic plants were obtained as described in Salazar-Iribe et al. [19].

Studies carried out to determine the subcellular localization of BDX in epidermal root cells of Arabidopsis suggested that it was localized intracellularly, probably in Golgi, and then relocated to the cell wall when the plants were subjected to abiotic (NaCl) or biotic (nematodes) stress stimuli [30] (Figure S2). *TEB* is highly expressed during cell division prior to cytokinesis. In Arabidopsis, the synchronization of mitosis in primary root cells treated with hydroxyurea [31] confirmed that TEB was localized intracellularly during mitosis, and was located in the cell wall until the end of the process (Figure 3). These results suggest that BDX and TEB are located transiently in the cell wall in response to endogenous or exogenous stimuli. Finally, polar localization of TEB in the cell wall of radicular hair was determined during its elongation, suggesting the formation of domains with an accumulation of these proteins within the cell walls [19].

## 4. Function of DUF642 Proteins in Plant Development

The expression patterns of *DUF642* genes suggest they are involved throughout the development and growth of plants. In general, changes in the expression patterns of *DUF642* genes can be related directly to PME activity [13,15,16,19]. The leaves of Arabidopsis lines overexpressing *AHDGR2* had low PME activity, and this was reflected in alterations in the pattern of de-methyl-esterified HGs in the cell walls [16,19,32].

### 4.1. Seed Development

In Arabidopsis, *BDX*, *At5g11420*, *TEB*, and *At3g08030* were expressed during embryo development (Table 1 and Table S1), and the localized expression during development indicated differential expression in different cell types (Figure 1). However, the *bdx-1*, *At2g11420*, and *teb-1* mutants did not show alterations in gene expression during embryo development.

During endosperm development, *BDX* was the only *DUF642* gene reported at four days after pollination [33]. Of the *dgr1*, *bdx-1*, *At5g11420*, *dgr2*, and *teb-1* mutants studied during seed development, only the *bdx-1* mutant showed alterations, producing misshapen seeds with changes in the folding of the embryo. The endosperm and seed coat exert physical restrictions during elongation and folding of the embryo [34], with the walls of the endosperm adjacent to the embryo showing a decrease in the degree of HGs esterification. In the *bdx-1* mutant, a lower signal of de-methyl-esterified HGs compared with the wild-type was detected in this region. Alterations in the degree of esterification can cause greater rigidity of the cell wall of endosperm cells, thereby generating a physical restriction in the development of the embryo and compromising the subsequent survival of the seedling [32].

The accumulation of *DUF642* gene transcripts has also been reported in mature seeds. Germination pre-treatments such as priming, which improve germination performance, can induce the expression of these genes. An important increase in the expression of the orthologous gene of *At3g08030* was reported in seeds from *Brassica oleracea* subjected to an osmopriming treatment [35]. In Arabidopsis, *Ceiba aesculifolia*, and *Wigandia urens* seeds, the expression of *At3g08030* increased in response to osmopriming treatment and decreased when the seeds were subjected to controlled deterioration [36]. In a study carried out with Arabidopsis *vps29* mutant seeds (VPS29 rearrangement complex), which had a lower germination index, the abundance of *At3g08030* and *At1g29980* increased while that of *At5g11420* and *DGR2* decreased [37]. Functional studies of *At3g08030* loss of function mutants could reveal if this gene has a role in promoting seed longevity.

After seed maturation, the expression of *At5g11420*, *DGR2*, *At3g08030*, and *At1g29980* increased in after-ripened seeds (Table S1). These changes in expression may be related to changes in the biochemical machinery that are required to modify the architecture of the cell wall of the embryo, the endosperm, and the seed coat during germination.

### 4.2. Germination

During the germination process of barley, Brassica, and Arabidopsis seeds, progressive differential expression of several *DUF642* genes took place (Table S1). The expression levels of Clade A1 genes (*BDX*, *At5g11420* and *DGR2*) and *At3g14310*, which codes for PME3, increased after 6 h of seed imbibition. The expression of the Clade A2 gene, *TEB*, increased between 8 and 12 h, and *At3g08030* expression was detected in the mature seeds and during the entire imbibition process (Figure S3).

The lines overexpressing *BDX*, *At5g11420*, and *DGR2* showed a decrease in the onset of germination, especially in the timing of the testa rupture, compared with the wild-type seeds [13] (Figure S3). The increase in total PME activity during seed germination in the overexpressing plants may be related to the early testa rupture described previously [14]. Early testa rupture also was observed in tomato seeds from tomato plants overexpressing *VqDUF642* [15]. These results indicate that the Clade A1 genes have the same function during germination.

### 4.3. Hypocotyls

Under control growth conditions (long photoperiod), the Arabidopsis *bdx-1* and *teb-1* mutant lines had longer hypocotyls because of longer cell length compared with the wild-type, whereas the overexpressing lines produced shorter hypocotyls. The hypocotyls of the *bdx-1* and *teb-1* mutants had a higher signal for methyl-esterified HGs. Additionally, the *bdx-1* mutant accumulated more auxins in the epidermal cells than the wild-type [18]. During cell elongation in the hypocotyl, auxin signaling and cell wall modifications of epidermal cells were actively involved [38,39]. The accumulation of auxins in hypocotyl epidermal cells during cell elongation was facilitated by PIN transporters. In the *bdx-1* mutant, a change in the localization of PIN1 transporters was detected, possibly because of the degree of HGs esterification, as has been described for other tissues [40]. The *bdx* and *teb* mutants showed an altered hypocotyl phenotype, suggesting *DUF642* genes may be involved in regulating hypocotyl elongation during seedling development [41].

### 4.4. Leaves

The expression of *DGR1*, *BDX*, *At5g11420*, *DGR2*, *TEB*, *At2g41810*, *At3g08030*, *At2g34510*, and *At5g14150* was detected in rosette leaves of mature Arabidopsis plants close to flowering (Table S1). The *dgr1*, *bdx-1*, and *teb-1* mutants showed no alterations in the development of rosette leaves. The *dgr2* mutant had a smaller rosette, explained by its expression in leaf primordia [17]. The lines overexpressing *BDX*, *At5g11420*, and *TEB* showed no visible alterations in the leaves or in the size of the rosette [13,19]. No differences were found in the leaves of Arabidopsis plants overexpressing *AhDGR2* or tomato plants overexpressing *VqDUF642* [15,16]. The mechanisms that control leaf origin and growth are complex, because of the phenotypic plasticity that is present in leaves as a mechanism for adaptation to different environments [42]. The complexity of the regulation of leaf size and morphology, and the lack of alterations in the leaves of mutant lines suggest the *DUF642* genes are functionally redundant in leaves.

### 4.5. Reproductive Structures

**Figure 4:**
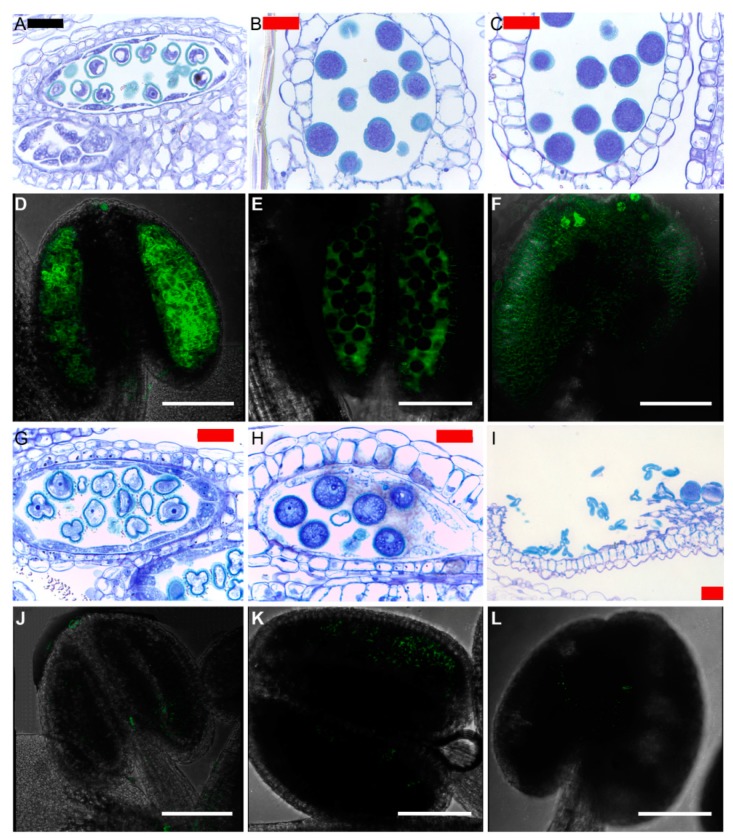
Pollen grain development and auxin accumulation in anthers of flowers from wild-type and *bdx-1* mutant Arabidopsis plants. Normal pollen grain development in wild-type (wt) flowers. (**A**) Anthers from 7–8 stage flower. (**B**) Anthers from 11–12 stage flower. (**C**) Anthers from 13 stage flower. GFP detection of auxins in anthers from wt*/DR5* flowers. (**D**) Anthers from 7–8 stage flower. (**E**) Anthers from 11–12 stage flower. (**F**) Anthers from 13 stage flower. Throughout the development of the anther there is an important accumulation of auxins. Pollen grain development in *bdx-1*/*DR5* flowers. (**G**) Anthers from 7–8 stage flower. (**H**) Anthers from 11–12 stage flower. (**I**) Anthers from 13 stage flower. The development of pollen grains is altered; at the 11–12 stage, diffuse cytoplasm is observed and, at stage 13, some pollen grains have collapsed. GFP detection of auxins in anthers from *bdx-1/DR5* flowers. (**J**) Anthers from 7–8 stage flower. (**K**) Anthers from 11–12 stage flower. (**L**) Fertilized flowers at stage 13. Throughout the development of the anther there is almost no accumulation of auxins. The Arabidopsis transgenic plants were obtained as described in Cruz-Valderrama et al. [32]. Sections: 1–2 μm. Staining was with toluidine blue. Red bar = 20 μm, Black bar = 10 μm. n = 8–10 plants for each line. White bar = 100 μm. n = 5–9 anthers from flowers from different plants.

During flower development in Arabidopsis, the *DUF642* genes studied to date showed differential expression patterns, although the expression of only two genes have been detected in carpel, stamens, and petals. *BDX* is the only *DUF642* gene that has been detected in the anthers of stamens. The *bdx-1* mutant line showed morphological alterations of the pollen grain that altered its viability. The in vitro germination of the pollen tube was lower in the *bdx-1* mutant pollen grains that in the wild-type. The structural and functional alterations of the pollen grains in the *bdx-1* mutant could be caused by changes in the accumulation of auxins during the late stages of anther development (Figure 4). Similar alterations in pollen morphology have been described in mutants related to the auxin signaling pathway, including auxin synthesis and transport [43].

### 4.6. Fruits

Studies of *DUF642* gene expression and gene function during fruit development are very scarce. Of the Arabidopsis mutants studied, only *bdx-1* showed a fruit phenotype with shorter siliques and lower seed production than wild-type plants. In the genus *Brassica*, *DGR2* is the only gene that has been detected in transcriptomes of siliques, specifically in the shells of the pods [44].

### 4.7. Roots

The expression of *DGR1*, *BDX*, *At5g11420*, *DGR2*, and *TEB* has been detected in primary roots of Arabidopsis. All these genes were expressed in the epidermal cells of the meristematic zone, except *DGR2*, which was expressed in the elongation zone. The *dgr2* mutant was the only one that presented a short root phenotype [17]. Overexpression of *AhDGR2* in Arabidopsis produced longer roots [16], which is understandable given that *DGR2* was expressed in the meristematic zone where the cells begin to expand and the rate of cell division is reduced.

The *bdx-1*, *At5g11420*, and *teb-1* mutants showed no alterations in the development of lateral roots. *BDX*, *At3g08030*, *At5g11420*, and *TEB* all had similar expression patterns throughout the development of lateral roots, which suggested there could be a functional redundancy that would explain the lack of altered phenotypes for these mutants.

Roots interact with microorganisms in the soil, and are particularly susceptible to pathogen attacks. Roots also are central in the perception of nutrient concentrations and other elements like heavy metals in the substrate where plants develop. The modification and restructuring of the cell wall in roots actively participate in the response to different types of stresses. Therefore, the participation of *DUF642* genes in the remodeling of the cell wall can be relevant in all these processes. Changes in the expression of *DUF642* genes in response to biotic and abiotic stresses have been described in various plant species (Table S2). However, very few functional studies are available related to the particular role of *DUF642* genes in response to stress.

## 5. Biotic Factors

*DGR2* is negatively regulated in Arabidopsis plants during infection by the bacterium *Ralstonia solanacerum* [45]. The expression of *At1g29980* and *At3g08030* is induced by infection with *Rhodococcus fascians* [46]. *At3g08030* also is highly expressed in response to infection by *Agrobacterium tumefaciens* [47].

The abundance of the DUF642 protein encoded by *At3g08030*, which has two isoforms with different isoelectric points, was reduced significantly in the cell wall proteome of Arabidopsis suspension cells treated with chitosan, an elicitor that mimics a fungal infection [48]. Infection with the *Penicillium* strain Pc4 in post-harvest grapes increased the abundance of two isoforms of the At5g11420 orthologous protein, probably because of post-translational modification [49]. In apoplastic proteomes of maize roots (*Zea mays*) infected with the symbiont species *Trichoderma virens*, a DUF642 protein was detected 5 days after inoculation. Modification and degradation of root cell walls are essential for colonization to take place [50]. Infection of Arabidopsis plants with the phytopathogenic fungus *Botrytis cinerea* reduced the expression of *At5g11420* and *At5g25460* [51].

The overexpression of *VqDUF642* in tomato plants reduced the susceptibility to *B. cinerea* infection in both mature and immature fruits. Overexpression of this gene modified the expression of some pathogen response genes such as *SIPR1*, *SIPR2*, *SIPR3*, and *SIPR4* whose expression increased at 48 h post-infection. In transgenic Thompson grape plants, overexpression of *VqDUF642* promoted increased resistance to *B. cinerea* and induced resistance to the fungus *Erysiphe necator,* which causes oidium of vines. The leaves of the overexpressing plants infected with *E. necator* showed a less severe infection than the wild-type at 48 h post-infection. In addition, the expression levels of the pathogen-response genes *VvPR1*, *VvPR2*, *VvPR3,* and *VvPR4* increased drastically at 48- and 96-h post-infection in transgenic seedlings compared with in the wild-type [15].

In Arabidopsis, transcriptomic induction during early inoculation with the knot-nematode *Meloidogyne incognita* revealed up-regulation of *At1g29980* [52]. *BDX* and *TEB* expression were also highly induced by *M. incognita* early inoculation. The cell wall localization of BDX and TEB in the epidermal cells of primary roots was induced by *M. incognita*. Early inoculation with *Nacobus aberrans*, a nematode that cannot infect Arabidopsis, did not alter the expression of these two genes [30].

A comparative analysis of available Arabidopsis flower transcriptomes showed that changes in the expression patterns of flower-specific defense genes were critical in pathogen resistance. According to this study, the expression of *BDX* and *DGR2* was positively regulated in petals compared with in senescent leaves, in stage 15 of the flower. At this stage, an increase in the expression of the biotic stress response genes also occurred [53].

## 6. Abiotic Factors

The regulation of the DUF642 genes by transcription factors in response to aluminum stress has been reported. Aluminum stress has been studied extensively in aluminum-resistant plants. In particular, *Oryza sativa* (rice) is a cultivated crop plant that is resistant to aluminum, and the signaling pathway associated with its resistance has been studied widely. The Al resistance transcription factor 1 gene (*ART1*) is central to the aluminum response, and it regulates an increase in the expression of the *DUF642* gene *Os04g049490* (orthologous to *At5g11420*) [54]. *Os04g049490* was also regulated by the *SENSITIVE TO ALUMINUM RHIZOTOXICITY* gene (*STAR1*), which plays a fundamental role in aluminum resistance in rice roots [55]. In the *O. sativa indica* IR64 cultivar treated with aluminum, *Os04g41750* (orthologous to *At5g11420*) expression was up-regulated, whereas no change in its expression was detected in *O. sativa* cv. Azucena, which is more sensitive to aluminum [56]. Plants in the genus *Stylosanthes* have high tolerance to toxicity by the aluminum ion, and expression of the orthologous *DUF642* gene *Os04g0494900* increased in the roots of the Reyan 2 genotype in response to aluminum treatment [57]. A comparison between the transcriptomes of two *Citrus* species with different aluminum tolerances suggested that the process of HGs de-esterification in the cell wall of the root cells played an important role in resistance to aluminum. In *C. sinensis*, which is resistant to aluminum, four genes related to this process and a *DUF642* gene, an ortholog of *DGR2*, were up-regulated [58]. In NtSTOP-1-RNAi tobacco plants that showed a decrease in the expression of the *SENSITIVE TO PROTON RHIZOTOXICITY* gene (*STOP1*), the expression of *Nt6860* (orthologous to *At5g11420)* decreased, as has been described for *At5g11420* in the Arabidopsis *art-1* mutant [59].

In *Medicago sativa* plants exposed to cadmium, the amount of the protein orthologous to the Arabidopsis protein, DGR2, decreased [60]. The exposure of *Populus* × *canadensis* plants to high doses of zinc promoted the decrease in the expression of *POPTR_0001s27110* (orthologous to *At3g08030*) [61].

Ultraviolet radiation (UV-B) generates an imbalance in the production of reactive oxygen species. Thus, it is used to study the effects of oxidative stress on plants. In the mutant *ggt1* of the protein GAMMA-GLUTAMYL TRANSFERASE ISOFORM 1(GGT1), involved in the redox balance, the proteins DGR2 and PECTIN METHYL ESTERASE3 (At3g14310) are very abundant in both the mutant and in the wt of Arabidopsis plants exposed to UV-B [62].

In the salinity-resistant plant *Manihot esculenta* Crantz (Cassava), *RknMes02_00171* and *RknMes02_00443*, which are orthologs of *BDX* and *At5g11420* respectively, were among the 40 most highly expressed genes during NaCl treatment [63]. *AhDGR2* was significantly induced in *Amaranthus hypochondriacus* (grain amaranth), which is also resistant to high doses of salt. However, when *AhDGR2* was overexpressed in Arabidopsis, the plants showed hypersensitivity to increasing NaCl concentrations, as shown by shorter root length, smaller and slightly chlorotic rosettes, as well as considerably reduced germination rates [16].

The expression levels of *At2g34510* and *At5g11420* increased in Arabidopsis plants subjected to drought stress, although their expression levels were not altered by treatment with abscisic acid [64]. The increase in *At2g34510* expression induced by drought was corroborated in a study that tested the stress response in natural populations of Arabidopsis [65]. In *Eucalyptus calmadulensis*, the expression of *Eucgr.C02812* (orthologous to *At5g11420*) was also found to decrease in plants exposed to drought [66].

## 7. Conclusions

The DUF642 proteins are highly conserved, which may be related to their specific interactions with cell wall polysaccharides and proteins in different cell types. The *At3g08030*-encoded protein interacts in vitro with cellulose, a polysaccharide that has an important role in plant development [9]. However, there are still no functional studies that confirm this interaction. BDX and At5g11420 interact in vitro with a PME [12]. Studies in different plant species suggest that their function could be related to the regulation of HGs modification throughout plant development. The functional studies carried out to date are consistent with this hypothesis. For instance, in almost all plant tissues where *DUF642* gene overexpression has been induced, there was an increase in the total PME activity. Furthermore, the increase in methyl-esterified HGs in the endosperm during embryo folding and in hypocotyl epidermal cells in the *DUF642* mutants is consistent with the corresponding phenotype. However, a direct interaction of DUF642 proteins with PMEs needs to be established using different experimental approaches. Several proteomic studies have indicated the presence of two isoforms of the same DUF642 protein, suggesting these proteins undergo post-translational modifications that may be related to differential interactions within the cell wall [29,60,61].

The different functions of *DUF642* genes during development can be determined by the specific spatial-temporal expression pattern for each gene. The differential response to hormones also may participate in this process. *BDX* is the only *DUF642* gene expressed in the endosperm during seed development. This specific expression may explain the misshapen-seeds phenotype in the *bdx-1* mutant. Similarly, the expression of *DGR2* in the root elongation zone may explain the short root phenotype exclusive of the *dgr2* mutant. It is possible that there is functional redundancy during embryo development as well as during the development and emergence of the lateral root, processes in which the same temporal-spatial pattern of at least two *DUF642* genes was detected in Arabidopsis.

*BDX*, *At5g11420*, *TEB*, and *At3g08030* are expressed in the meristematic zone of the primary root, but the *bdx* and *teb* mutants do not show an altered root phenotype. BDX was located intracellularly in the epidermal cells of the primary root and relocated to the cell wall in response to biotic and abiotic stresses. Roots interact with different organisms in the soil and are subjected to multiple perturbations in the environment, such as decreases in the availability of water and increases in toxic ions. These interactions can alter the expression of some DUF642 genes. In the response to fungal infection, the overexpression of *VqDUF642* increased resistance to the pathogen, although the expression other *DUF642* genes decreased in response to infection. These results suggest that the *DUF642* genes participate broadly in plant responses to environmental factors and not solely in the root developmental process.

The primary structure of *DUF642* proteins is highly conserved in different spermatophyte species. However, studies of their expression patterns in Arabidopsis showed that the spatial-temporal expression pattern for each gene was specific and consistent with the phenotypes of the mutant plants studied so far. The regulation of *DUF642* gene expression by hormones and environmental stimuli also was specific for each gene. Functional studies of the *DUF642* genes in different plant species are needed to determine the relevance of the DUF642 family in the evolution of terrestrial plants.

## Figures and Tables

**Table 1:** Expression profiles of the *DUF642* genes detected in distinct tissues/organs and species. *DUF642* genes are grouped by clades as reported by Vázquez-Lobo et al. (2012) [9] and using the locus tag of Arabidopsis as a reference for gene grouping.

Clade	Gene Name	Species	Expressed in Tissue/Organ	Reference
A1	*At1g80240*	Arabidopsis	Petal	[23]
A1	*GRMZM2G027683*	*Zea mays*	Embryo	[24]
A1	*OSNPB_040494600*	*Oryza sativa*	Floral buds, flowers, mature seed, milk grains, roots before flowering	[25]
A1	Orthologous to *At4g32460*	*Brassica rapa*	Hypocotyl epidermal cells	[21]
A1	Orthologous to *At4g32460*	*Cannabis sativa*	Stem (apical section)	[26]
A1	*OSNPB*_020205200	*Oryza sativa*	Floral buds, flowers, mature seed, milk grains	[25]
A1	Orthologous to *At5g11420*	*Brassica rapa*	Hypocotyl epidermal cells	[21]
A1	*At5g11420*	Arabidopsis	Petal	[23]
A1	*At5g11420*	Arabidopsis	Root (cortex)	[27]
A1	*OSNPB_010611000*	*Oryza sativa*	Floral buds, flowers, mature seed, milk grains, roots before flowering	[25]
A1	*OSNPB_040494800*	*Oryza sativa*	Floral buds, milk grains, roots before flowering	[25]
A1	Orthologous to *At5g25460*	*Brassica rapa*	Hypocotyl epidermal cells	[21]
A1	*AhDGR2*	*Amaranthus hypochondriacus* var. Revancha	Panicle, axillary bud, young stem, and young leaf	[16]
A1	*GRMZM2G051571*	*Zea mays*	Seed	[24]
A1	*GRMZM2G034985*	*Zea mays*	Seed, shoot, root, SAM, ear, tassel, cob, silk, anthers, ovule	[24]
A1	Orthologous to *At5g25460*	*Cannabis sativa*	Stem (apical section)	[26]
A1	*At5g25460*	Arabidopsis	Root (cortex)	[27]
A2	similar to *At3g08030*	*Brassica rapa*	Hypocotyl inner tissue cells	[21]
A2	*At3g08030*	Arabidopsis	Root (cortex/hair cell)	[27]
A2	*At2g41800*	Arabidopsis	Carpel	[23]
A2	*OSNPB_030807700*	*Oryza sativa*	Floral buds, flowers, mature seed, milk grains, roots before flowering	[25]
A2	*At2g41810*	*Brassica rapa*	Hypocotyl epidermal cells	[21]
B	Orthologous to *At1g29980*	*Brassica rapa*	Hypocotyl inner tissue cells	[21]
B	*OSNPB*_010756600	*Oryza sativa*	Floral buds, flowers, leaves before flowering, mature seed, milk grains, roots before flowering	[25]
B	*GRMZM2G324705*	*Zea mays*	Seed, shoot, root, SAM, ear, tassel, cob, silk, anthers, ovule	[24]

## Supplementary Materials

Supplementary materials can be found at https://www.mdpi.com/1422-0067/20/13/3333/s1. Figure S1. *At3g08030* expression during *Arabidopsis thaliana* development using *pAt3g08030::ER-GFP* plants; Figure S2. *At5g11420* expression during *Arabidopsis thaliana* development using *pAt5g11420::ER-GFP* plants; Figure S3. *At4g32460*/*BDX, At5g25460/DGR2*, *At5g11420*, *At3g14310/PME3, At2g41800/TEB* and *At3g08030* expression during seed germination; Figure S4. Subcellular localization of BDX in *Arabidopsis thaliana* primary roots under salinity stress conditions using *pBDX::BDX-GFP* plants. Table S1. Transcriptomic evidence of *DUF642* genes expression during specific developmental stages and/or in response to diverse environmental stimuli. Table S2. Transcriptomic evidence of *DUF642* genes expression in response to diverse environmental stimuli.
