# Supplementary material for: Overview of the Role of Cell Wall DUF642 Proteins in Plant Development
Source: Int J Mol Sci. 2019 Jul 6;20(13):3333. doi: 10.3390/ijms20133333 (PMC6651502; doi:10.3390/ijms20133333)
Supplement: Supplementary file 1 [file ijms-20-03333-s001.zip › Supplemental_material-v5.pdf]

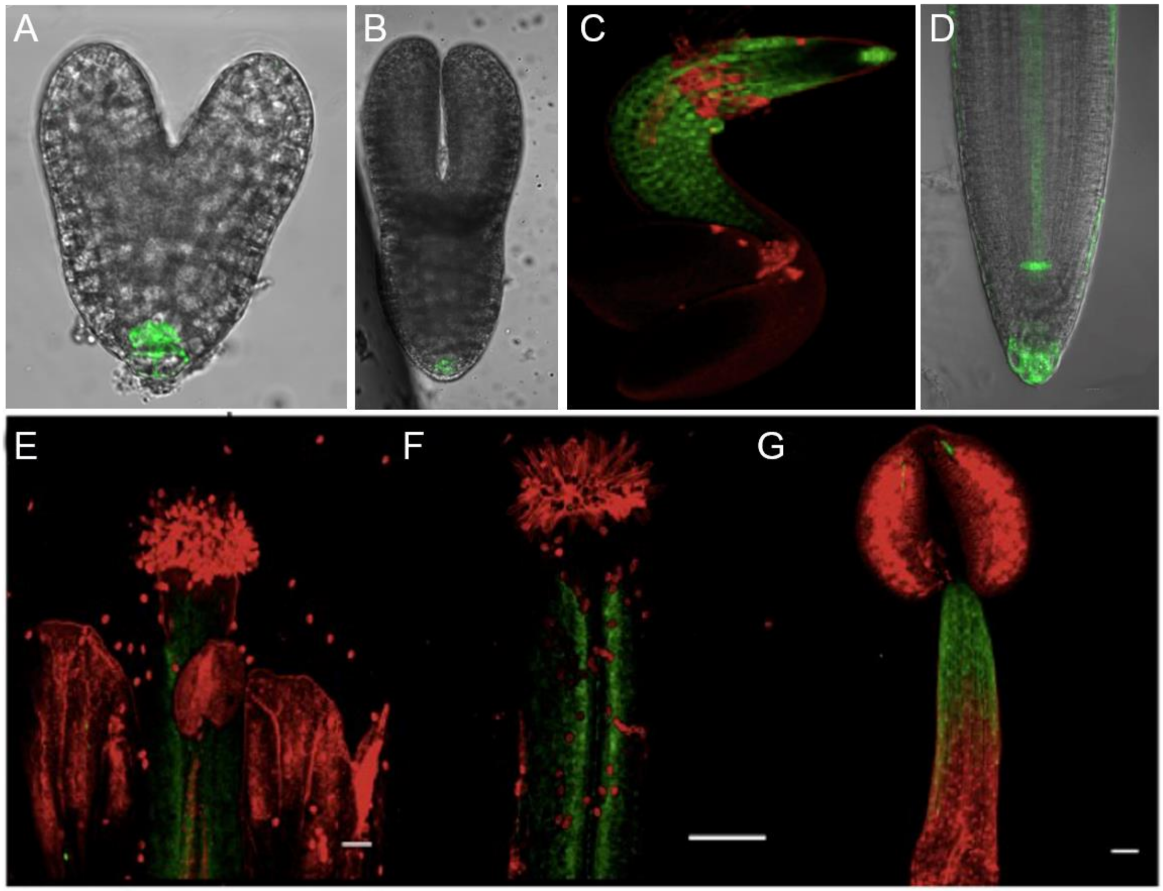

Figure S1. *At3g08030* expression during *Arabidopsis thaliana* development using *pAt3g08030::ER-GFP* plants. A) GFP fluorescence in embryo at heart stage. B) GFP fluorescence in embryo at torpedo state. C) GFP fluorescence in hypocotyl and primary root of a 36-h germinating seed. D) GFP fluorescence in the primary root from 7-d seedlings. E) GFP fluorescence in flower. F) GFP fluorescence in carpel and G) GFP fluorescence in stamen. *pAt3g08030::ER-GFP* transgenic plants were obtained and laser confocal scanning microscopy was done as described in Salazar-Iribe *et al.* [19]. ER-GFP refers to Endoplasmic Reticulum signal peptide fused to GFP, A, B, D are longitudinal sections and C, E, F and G are projections of confocal stacks.

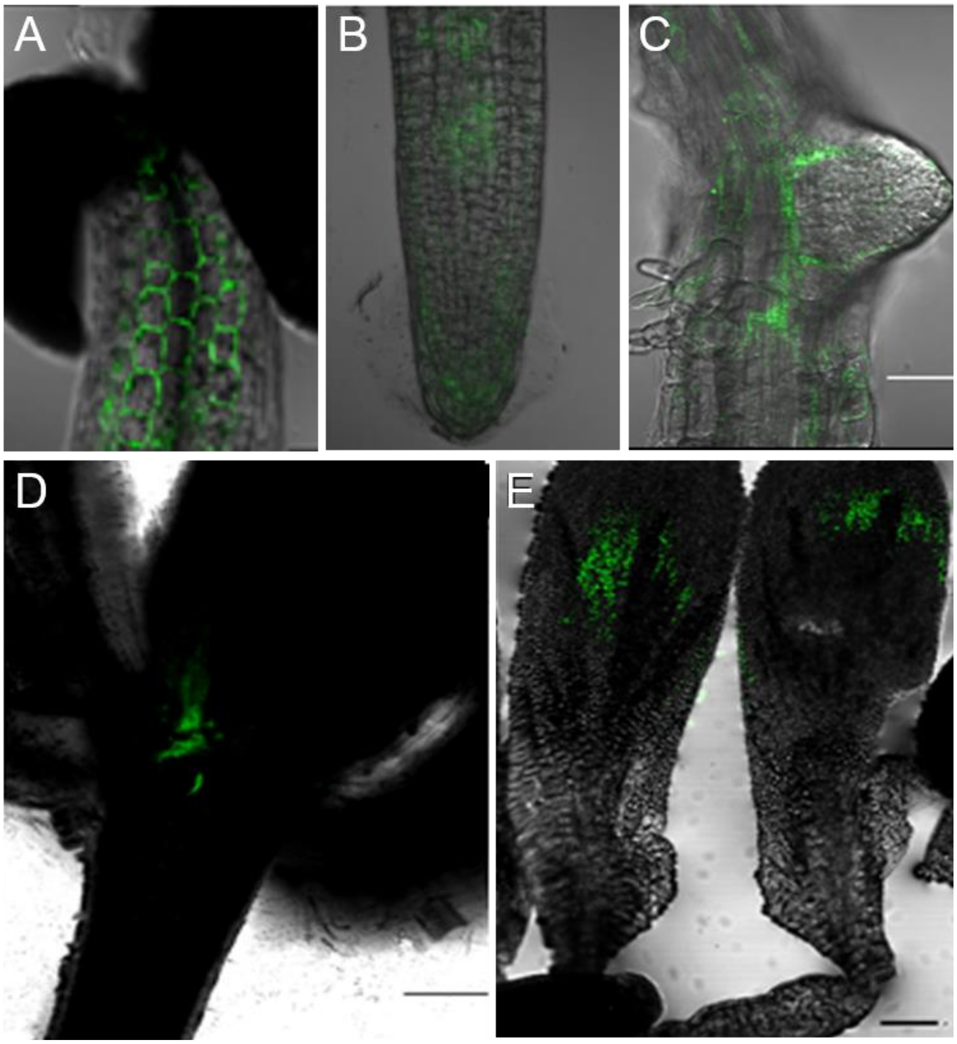

Figure S2. *At5g11420* expression during *Arabidopsis thaliana* development using *pAt5g11420::ER-GFP* plants. A) GFP fluorescence in hypocotyl of a 48-h germinating seed. B) GFP fluorescence in the primary root from 7-d seedlings. C) GFP fluorescence during lateral root emergence. D) GFP fluorescence in flower. E) GFP fluorescence in petals *pAt5g11420::ER-GFP* transgenic plants were obtained and laser confocal scanning microscopy was done as described in Zúñiga-Sánchez *et al.* [13]. Images are projections of confocal stacks.

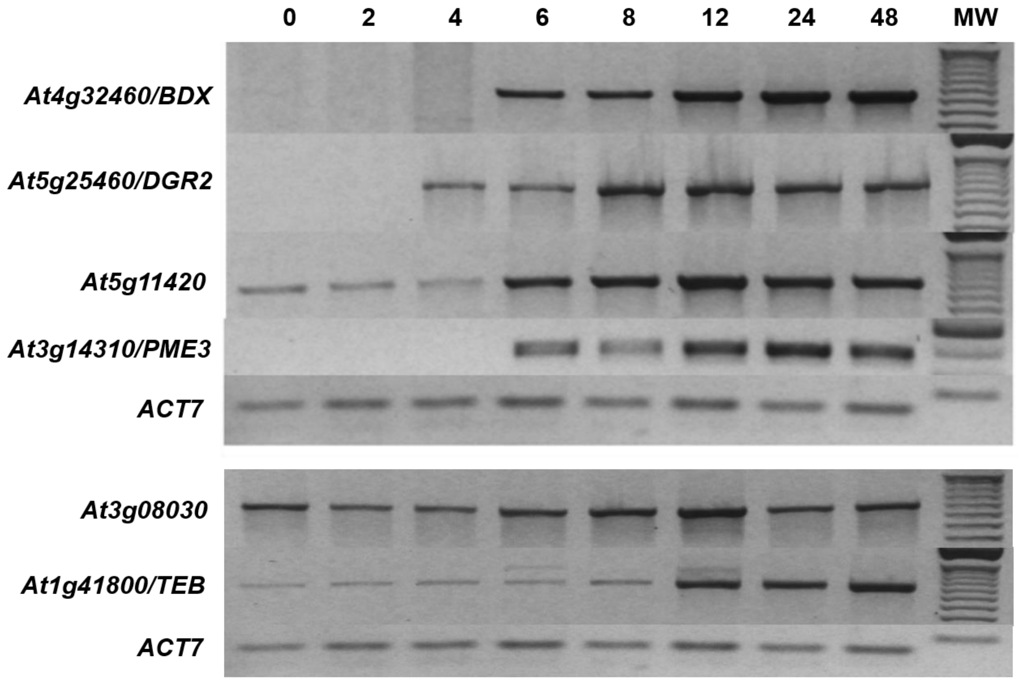

Figure S3. *At4g32460/BDX, At5g25460/DGR2* , *At5g11420*, *At3g14310/PME3* , *At2g41800/TEB* and *At3g08030* expression during seed germination. Seeds were collected after 2, 4, 6, 8, 12, and 24 h of imbibitions. 0 corresponds to dry seed and 48 to germinated seeds. *ACT7* expression was used as control. Primers used: BDXF 5´GTGATAGTGCTTCTTCTCCTTCAC 3´; BDXR 5´AGCGACGAATCTCAATGAC 3´; DGR2F 5´CTTCCTTCTTTTCATCGCC 3´; DGR2R 5´ACGAGAAATCATCGCTCC 3´; At5g11420F 5´AATCGCCACCATCACTTC3´; At5g11420R 5´ CATAACACTTGTGCGGGTC; 3´At3g08030F 5´GGTTCCCAAAGCCATTATTC 3´; At3g08030R 5´ ACAATCTCGTCAATGACAGG 3´; ´TEBF 5´TCCTCCTCCTATCTCTCTGC 3´; TEBR 5´AAACGGTTCTCTTCCTGC 3´; PMEF 5´CATCAATGAAAGAAATTTTTTC 3; PMER 5´AGACCGAGCGAGAAGGGGAAA 3´; ACT7F 5´GGTCGTACAACCGGTATTGT 3´; ACT7R 5´GAAGAGCATACCCCTCGTA 3´. PCR analyses were done as described in Garza-Caligaris *et al.* [36].

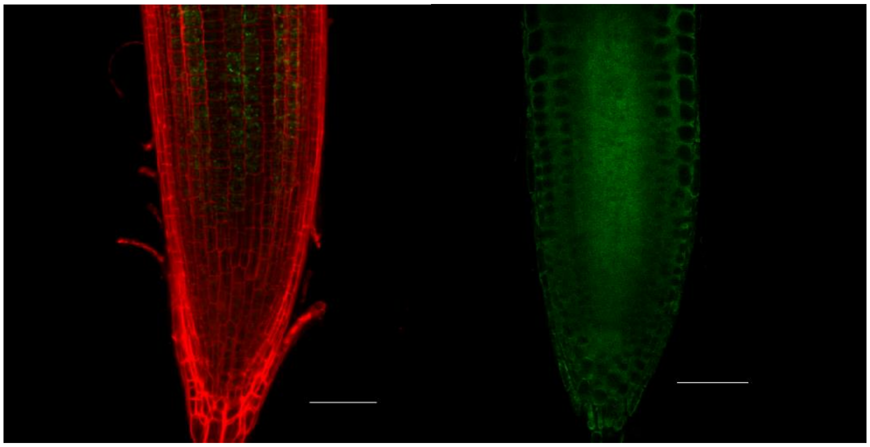

Figure S4. Subcellular localization of BDX in *Arabidopsis thaliana* primary roots under salinity stress conditions using *pBDX::BDX-GFP* plants. A) GFP fluorescence is detected intracellulary in the epidermal cells of the primary root from seedlings grown under control conditions. B) GFP fluorescence is detected in the cell wall of the epidermal cells o f the primary toot of seedlings grown under salinity stress.

Seedling were grown in MS with 50 mM NaCl. *pBDX::BDX-GFP* plants transgenic plants were obtained and laser confocal scanning microscopy was done as described in Salazar-Iribe *et al.* [30]. A is a longitudinal section and B is a projection of confocal stacks.

References for Supplemental material

67. Dekkers, B.J.; Pearce, S.; van Bolderen-Veldkamp, R.P.; Marshall, A.; Widera, P.; Gilbert, J.; Drost, H.G.; Bassel, G.W.; Müller, K.; King, J.R.; et al. Transcriptional dynamics of two seed compartments with opposing roles in Arabidopsis seed germination. *Plant Physiol.* **2013**, *163*, 205–215.
68. Carrera, E.; Holman, T.; Medhurst, A.; Dietrich, D.; Footitt, S.; Theodoulou, F.L.; Holdsworth, M.J. Seed after-ripening is a discrete developmental pathway associated with specific gene networks in Arabidopsis. *Plant J.* **2008**, *53*, 214–224.
69. Le, B.H.; Cheng, C.; Bui, A.Q.; Wagmaister, J.A.; Henry, K.F.; Pelletier, J.; Kwong, L.; Belmonte, M.; Kirkbride, R.; Horvath, S.; et al. Global analysis of gene activity during Arabidopsis seed development and identification of seed-specific transcription factors. *Proc. Natl. Acad. Sci. USA* **2010**, *107*, 8063–8070.
70. Sreenivasulu, N.; Usadel, B.; Winter, A.; Radchuk, V.; Scholz, U.; Stein, N.; Weschke, W.; Strickert, M.; Close, T.J.; Stitt, M.; et al. Barley grain maturation and germination: Metabolic pathway and regulatory network

commonalities and differences highlighted by new MapMan/PageMan profiling tools. *Plant Physiol.* **2008**, *146*, 1738–1758.
71. Tung, C.W.; Dwyer, K.G.; Nasrallah, M.E.; Nasrallah, J.B. Genome-wide identification of genes expressed in Arabidopsis pistils specifically along the path of pollen tube growth. *Plant Physiol.* **2005**, *138*, 977–989.
72. Dekkers, B.J.; Pearce, S.P.; van Bolderen-Veldkamp, R.P.M.; Holdsworth, M.J.; Bentsink, L. Dormant and after-ripened Arabidopsis thaliana seeds are distinguished by early transcriptional differences in the imbibed state. *Front. Plant Sci.* **2016**, *7*, 1323.
73. Palovaara, J.; Saiga, S.; Wendrich, J.R.; van‘t Wout Hofland, N.; van Schayck, J.P.; Hater, F.; Mutte, S.; Sjollema, J.; Boekschoten, M.; Hooiveld, G.J.; et al. Transcriptome dynamics revealed by a gene expression atlas of the early Arabidopsis embryo. *Nat. Plants* **2017**, *3*, 894.
74. Cadman, C.S.; Toorop, P.E.; Hilhorst, H.W.; Finch-Savage, W.E. Gene expression profiles of Arabidopsis Cvi seeds during dormancy cycling indicate a common underlying dormancy control mechanism. *Plant J*. **2006**, *46*, 805–822.
75. de Simone, A.; Hubbard, R.; de la Torre, N.V.; Velappan, Y.; Wilson, M.; Considine, M.J.; Soppe, W.J.; Foyer, C.H. Redox changes during the cell cycle in the embryonic root meristem of *Arabidopsis thaliana*. *Antioxid. Redox Signal.* **2017**, *27*, 1505–1519.
76. Nelson, D.C.; Flematti, G.R.; Riseborough, J.A.; Ghisalberti, E.L.; Dixon, K.W.; Smith, S.M. Karrikins enhance light responses during germination and seedling development in *Arabidopsis thaliana*. *Proc. Natl. Acad. Sci*. *USA* **2010**, *107*, 7095–7100.
77. Hofmann, F.; Schon, M.A.; Nodine, M.D. The embryonic transcriptome of *Arabidopsis thaliana*. *Plant Reprod*. **2019**, *32*, 77–91.
78. Van Veen, H.; Vashisht, D.; Akman, M.; Girke, T.; Mustroph, A.; Reinen, E.; Hartman, S.; Kooiker, M.; van Tienderen, P.; Schranz, M.E.; et al. Transcriptomes of eight *Arabidopsis thaliana* accessions reveal core conserved, genotype-and organ-specific responses to flooding stress. *Plant Physiol.* **2016**, *172*, 668–689.
79. Reem, N.T.; Chen, H.Y.; Hur, M.; Zhao, X.; Wurtele, E.S.; Li, X.; Li, L.; Zabotina, O. Comprehensive transcriptome analyses correlated with untargeted metabolome reveal differentially expressed pathways in response to cell wall alterations. *Plant Mol. Biol.* **2018**, *96*, 509–529.
80.
81. Kawa, D.; Julkowska, M.M.; Sommerfeld, H.M.; Ter Horst, A.; Haring, M.A.; Testerink, C. Phosphate-dependent root system architecture responses to salt stress. *Plant Physiol.* **2016**, *172*, 690–706.

82. Watson-Lazowski, A.; Lin, Y.; Miglietta, F.; Edwards, R.J.; Chapman, M.A.; Taylor, G. Plant adaptation or acclimation to rising CO_2? Insight from first multigenerational RNA-Seq transcriptome. *Glob. Chang. Biol.* **2016**, *22*, 3760–3773.

83. Kreps, J.A.; Wu, Y.; Chang, H.S.; Zhu, T.; Wang, X.; Harper, J.F. Transcriptome changes for Arabidopsis in response to salt, osmotic, and cold stress. *Plant Physiol.* **2002**, *130*, 2129–2141.
